# A Curious Case of Galactorrhea in the Context of Gastroenteritis: A Case Report

**DOI:** 10.7759/cureus.100679

**Published:** 2026-01-03

**Authors:** Ana T Baía, Rita Santos Alves, Catarina F Fonseca, Carla Martins

**Affiliations:** 1 Department of Family Medicine, Unidade de Saúde Familiar de São Marcos, Unidade Local de Saúde Amadora-Sintra, Sintra, PRT

**Keywords:** adverse drug reaction, galactorrhea, gastroenteritis, metoclopramide, primary care, quaternary prevention

## Abstract

Metoclopramide is a prokinetic and antiemetic agent widely used in primary care. Its mechanism of action explains both its therapeutic effects, as well as certain adverse reactions, including hyperprolactinemia and galactorrhea. Metoclopramide-induced galactorrhea may prompt concern and lead to unnecessary diagnostic testing.

This case report is about a healthy 21-year-old woman taking a combined oral contraceptive who was prescribed metoclopramide for gastroenteritis with vomiting. Forty-eight hours after starting treatment, she developed spontaneous, bilateral, multiductal, painless galactorrhea without menstrual changes, headaches, neurological symptoms, or signs of pregnancy. Physical examination confirmed bilateral milk secretion without inflammatory signs or palpable masses, and the neurological exam was normal. Given the clear temporal relationship and absence of alternative causes, metoclopramide was discontinued. At follow-up, complete resolution of symptoms occurred, supporting the diagnosis of drug-induced galactorrhea.

This case underscores an adverse reaction associated with a commonly used medication in Family Medicine. Clinicians should remain vigilant for the early identification of potential adverse drug reactions and avoid unnecessary diagnostic testing while maintaining a person-centered approach in routine Family Medicine practice.

## Introduction

Galactorrhea is defined as milk secretion from the breast outside pregnancy or breastfeeding [1,2]. It may arise as a manifestation of multiple conditions, including pituitary disorders, thyroid dysfunction, or as a side effect of medications (among these, antidopaminergic agents) [1]. The evaluation of galactorrhea should include a detailed medication history, obstetric history, assessment of galactorrhea, neurological symptoms, menstrual abnormalities, and systemic symptoms. Essential laboratory tests include measurement of serum prolactin, thyroid-stimulating hormone (TSH), and renal function. Pituitary MRI is indicated in cases of persistent hyperprolactinemia, neurological symptoms, or markedly elevated prolactin levels [3].

Although the mechanism by which the latter induces galactorrhea is well established - essentially through inhibition of dopaminergic action on the anterior pituitary, resulting in elevated prolactin levels - this effect is often underestimated in clinical practice. Such underrecognition may lead to extensive and sometimes unnecessary investigations (particularly in self-limiting and short-duration cases) [4]. In this context, it is important to highlight the concept of “quaternary prevention.” This principle, grounded in *primum non nocere* (first, do no harm), aims to identify individuals at risk of overmedicalization, protect them from dispensable diagnostic or therapeutic procedures, and avoid iatrogenesis [5]. Implementing quaternary prevention requires critical appraisal of the available evidence; that is, each preventive intervention or diagnostic and therapeutic method must demonstrate a clear benefit outweighing potential risks [6]. 

Metoclopramide, a drug with prokinetic and antiemetic properties, is widely used in primary and hospital care. Its most common indications include the treatment of nausea and vomiting (including during pregnancy), prophylaxis of chemotherapy- or postoperative-induced nausea and vomiting, paralytic ileus, gastroesophageal reflux disease after failure of standard therapy, and gastroparesis associated with diabetes mellitus [7]. Its central antidopaminergic effect (central and peripheral D₂-receptor blockade) [8-12] explains not only its gastrointestinal actions - enhanced motility of the upper digestive tract, accelerated gastric emptying, and increased lower esophageal sphincter tone - but also adverse effects related to hyperprolactinemia [10].

The United States Food and Drug Administration (FDA) recognizes that metoclopramide may raise prolactin levels and cause galactorrhea, amenorrhea, gynecomastia, and impotence - effects stemming from suppression of the hypothalamic-pituitary-gonadal axis [10]. Clinical reports show that metoclopramide-induced galactorrhea typically appears within weeks after initiation of therapy, resolving after drug discontinuation with subsequent normalization of prolactin levels [13,14].

The most common adverse effects include restlessness, drowsiness, fatigue, and lassitude. Among severe neurological effects, extrapyramidal reactions (dystonia, parkinsonism, and akathisia), tardive dyskinesia, depression, neuroleptic malignant syndrome, and hyperprolactinemia are notable [10,11]. Although galactorrhea is less prevalent than other neurological effects, its occurrence is clinically relevant [10]. From a regulatory standpoint, the European Medicines Agency (EMA) restricts the use of metoclopramide to a maximum of five days due to neurological risk, and the FDA maintains a boxed warning for tardive dyskinesia with prolonged treatment [14].

Metoclopramide-induced galactorrhea results from increased prolactin secretion (hyperprolactinemia) [8,9], an effect documented in both adult and pediatric populations and associated with inhibition of dopamine-mediated suppression of prolactin release from the anterior pituitary [11].

Since the 1980s, metoclopramide has been investigated as a galactagogue because of its dopamine-inhibiting effect and associated transient increase in serum prolactin; however, current evidence does not support its clinical efficacy. Although some studies demonstrate prolactin elevation, randomized trials and meta-analyses show no meaningful increase in breast milk volume compared with placebo, including among mothers of preterm infants, while domperidone has shown greater effectiveness in this subgroup. Major professional organizations concur that pharmacological galactagogues, including metoclopramide, should not be first-line therapy due to limited benefit and potential adverse effects, recommending instead that management of hypogalactia focus on non-pharmacological measures, such as specialized breastfeeding support, optimization of technique, frequent and effective milk removal, and correction of reversible factors [15-20].

This article was previously presented as a poster at the III Encontro Ibérico de Medicina Preventiva da Guarda in Portugal on November 7, 2025.

## Case presentation

A 21-year-old woman with no relevant personal or family medical history, and regularly taking a combined oral contraceptive (ethinylestradiol + levonorgestrel) for the past two years, presented to the Emergency Department with a two-day history of nausea and vomiting. She denied fever, diarrhea, severe abdominal pain, or ingestion of suspicious foods. She had no prior history of gastrointestinal, endocrine, or neurological disease.

At the initial physical examination, the patient was conscious, oriented, afebrile, and hemodynamically stable (blood pressure 118/74 mmHg; heart rate 84 beats per minute; oxygen saturation 99% on room air). The abdomen was soft and non-tender, with no organomegaly or signs of peritoneal irritation. No neurological abnormalities or significant signs of dehydration were noted. Given the diagnosis of probable acute viral gastroenteritis, symptomatic treatment was initiated with an antiemetic (metoclopramide, 10 mg orally, prescribed 30 minutes before the three main meals - breakfast, lunch, and dinner), along with dietary advice and oral hydration measures.

Forty-eight hours after leaving the Emergency Department, and after five doses of metoclopramide, 10 mg, the patient presented to her primary healthcare center reporting the sudden onset of spontaneous bilateral breast discharge. The patient denied mastalgia, breast swelling, skin changes, or a history of local trauma. She also denied menstrual irregularities, symptoms of hyperprolactinemia (amenorrhea, infertility, decreased libido), or neurological symptoms (headache, visual disturbances, vertigo, or seizures). The patient confirmed correct and consistent use of oral contraception, without missed pills.

At the second physical examination, the general condition was preserved, with no warning signs. On breast examination, a spontaneous and expressible bilateral milky discharge was observed, bilateral, multiductal, of moderate intensity, and visible on the underwear. No nodules, skin retraction, areolar changes, or inflammatory signs were detected. The summary neurological examination did not reveal motor or sensory deficits; the visual fields were apparently preserved, and cranial nerve functions were intact.

For differential diagnosis, laboratory testing was ordered to evaluate thyroid function, prolactin levels, and beta human chorionic gonadotropin (β-HCG). Considering the close temporal relationship between metoclopramide administration and symptom onset, as well as the absence of other evident causes of hyperprolactinemia, the medication was discontinued immediately, and a short-term follow-up appointment was scheduled. At reassessment one week after the primary care evaluation, the patient was asymptomatic, with complete resolution of the breast discharge. Laboratory results were within normal ranges (negative β-HCG; TSH, free thyroxine (T4); serum prolactin within reference values).

Causality assessment using the WHO-UMC system classified the adverse reaction as “Probable,” based on a plausible temporal relationship, exclusion of alternative causes, and complete resolution after drug withdrawal. Although symptom onset was earlier than typically reported, metoclopramide’s known dopamine D2 antagonism and prolactin-elevating effect support biological plausibility; rechallenge was not performed for ethical reasons, precluding a “Certain” classification. The probable diagnosis was established as metoclopramide-induced galactorrhea, which resolved after discontinuation of the medication.

**Figure 1:**
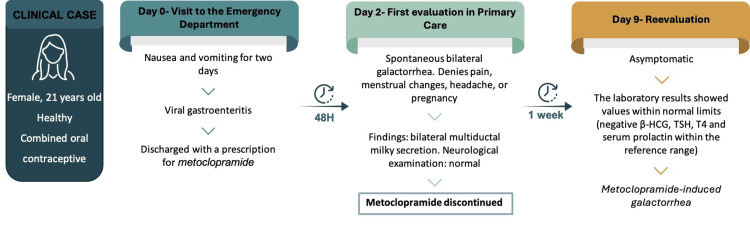
Timeline of metoclopramide-induced galactorrhea, showing onset within 48 hours of treatment initiation and complete resolution after drug discontinuation. β-HCG, beta human chorionic gonadotropin; TSH, thyroid-stimulating hormone; T4, free thyroxine

Figure 1 presents an infographic summarizing the case report, highlighting its key points and critical moments.

## Discussion

Metoclopramide-induced galactorrhea is a recognized but often underreported adverse effect, resulting from D₂ receptor blockade in the anterior pituitary and consequent hyperprolactinemia [8-10]. In this case, the close temporal relationship between metoclopramide administration and the onset of spontaneous bilateral galactorrhea, along with rapid resolution after drug discontinuation, strongly supports a causal association.

This report emphasizes the importance of careful clinical evaluation and rational diagnostic reasoning. In an otherwise healthy young woman with normal menstrual cycles, no neurological signs, and an unremarkable breast examination, extensive hormonal or imaging workup may be unnecessary. The temporal relationship strongly suggested drug-induced galactorrhea; baseline investigations were performed to provide patient reassurance, establish documented baseline values for medico-legal purposes, definitively exclude pregnancy, and ensure no coincidental pathology. This selective investigation represented appropriate quaternary prevention, as more extensive workup (pituitary MRI, prolonged prolactin monitoring, provocative testing) was appropriately avoided.

Overuse of tests, pharmacological therapies, and unnecessary clinical interventions can generate anxiety and constitute a source of resource consumption, potential complications, and avoidable costs for both the patient and the healthcare system. The family doctor plays a key role in avoiding unnecessary investigations and promoting a “wait-and-see” approach when justified, as well as in health education, shared decision-making, and the early identification of adverse drug reactions, enabling efficient and safe clinical management. Recognizing drug-related causality and prioritizing observation aligns with principles of quaternary prevention, minimizing patient anxiety, unnecessary tests, and iatrogenic risk [5].

In this case report, the family doctor played a crucial role in the early identification of a potential adverse drug reaction by correlating the timing of prescribed therapy with subsequent symptoms and excluding secondary causes of galactorrhea; in clinical monitoring and the early detection of symptoms suggestive of galactorrhea, thereby avoiding unnecessary complementary diagnostic procedures, such as extensive imaging, when a temporal relationship with the drug is evident and no other alarm signs are present; in the immediate discontinuation of the implicated medication and close clinical follow-up, promoting symptom resolution and preventing future complications; and in effective communication with the patient, providing clarification regarding the benign and reversible nature of the condition, thereby reducing associated anxiety and promoting health literacy.

## Conclusions

Metoclopramide-induced galactorrhea, though uncommon, represents a clinically significant adverse effect that may be underestimated in routine practice. This case underscores the importance of recognizing the temporal association between drug initiation and symptom onset, thereby preventing unnecessary and potentially iatrogenic investigations, in accordance with the principles of quaternary prevention. Given the widespread use of metoclopramide as an antiemetic and prokinetic agent, its adverse effect profile, particularly the risk of hyperprolactinemia and related manifestations, requires vigilant clinical monitoring.

Family doctors play a pivotal role in the early identification of medication-related adverse reactions, the judicious evaluation of the need for complementary investigations, and timely decisions regarding drug discontinuation. A person-centered approach, grounded in careful clinical observation, prudent interpretation of evidence, and clear communication, can minimize patient anxiety, avoid unnecessary procedures, and promote safe resolution. Nonetheless, the findings are limited by the report of a single case involving a young, otherwise healthy woman and short-term drug exposure, which restricts generalizability to other populations, comorbid conditions, and longer treatment durations.
